# Impact of chest pain protocol in the use of fibrinolytic therapy in a private hospital network with access to telemedicine in a middle income country

**DOI:** 10.1186/cc14663

**Published:** 2015-09-28

**Authors:** Pedro Gabriel MB Silva, Antonio Baruzzi, Giuliano Generoso, Henrique Ribeiro, Jose Carlos Teixeira, Marcelo Jamus, Mariana Y Okada, Sheila Simoes, Thiago A Macedo, Valter Furlan

**Affiliations:** 1 Hospital Totalcor, Cerqueira Cesar, São Paulo, São Paulo, Brazil

## Introduction

Brazilian registries [1] have shown that there is a gap between evidence-based therapies and the real treatment provided to patients with myocardial infarction. A chest pain protocol was implemented in a private hospital group in 2012 aiming at standardized optimal care for these patients.

## Objective

To evaluate the hypothesis of improving the use of reperfusion therapy and benefit in clinical outcomes in patients with STEMI after 2 years of implementation of the protocol in a large chest pain network.

## Methods

In 2012, physicians and nurses from 22 emergencies were trained to comply with a chest pain protocol and were provided access to telemedicine with a reference cardiologist available 24 hours a day, 7 days a week, for clinical discussion. All cases of ST segment elevation myocardial infarction (STEMI) were transferred to a reference hospital and the use of fibrinolytics before transfer (pharmacoinvasive strategy) was recommended. Data of STEMI patients transferred in 2011 (before protocol and telemedicine) were compared with the patients treated in 2013/14 (after implementation). A maximum limit of significance of 5% was defined for the chance of type I error (p < 0.05 was considered statistically significant).

## Results

The number of patients transferred to the reference hospital was 113 (2011), 140 (2013) and 123 (2014). Fibrinolytic therapy was used in 43 patients (38%) in 2011 and in 147 cases (55.8%) in 2013/14 (*p *= 0.002). The mortality rates of transferred STEMI cases were 8% in 2011 (nine cases) and 3% (eight cases) in 2013/14 (*p *= 0.06) with an observed/expected mortality by Grace score of 1.11 (95% CI, 0.39-1.83) in 2011 and 0.68 (95% CI, 0.21-1.15) in 2013/14. Along the years of 2011, 2012 and 2013, two patients treated with fibrinolytics died during the hospital stay (1.05%), whereas hospital mortality was 8.06% among those not treated with thrombolysis (p < 0.001). The patients that received reperfusion therapy in the first hospital used telemedicine more frequently (63.3% versus 42.2%; p = 0.001). See Table 1.

**Table 1:** Baseline characteristics.

	2011 (*n *= 113)	2013/14 (*n *= 263)	*p *value
Mean age (years)	59.6 (±13)	58.7 (±11)	0.49
Male	70%	74%	0.47
Hypertension	62%	64%	0.81
Diabetes	26%	29%	0.61
Previous PCI or CABG	14.2%	10.6%	0.43
Previous myocardial infarction	7.1%	5.7%	0.78
Mean Grace risk score (mortality)	7.2%	4.4%	0.5

*PCI *percutaneous coronary intervention, *CABG *coronary artery bypass graft.

## Conclusion

Two years after implementation of a chest pain protocol in a private emergency network, there was significant increase in the use of reperfusion therapy probably explained by a more frequent use of telemedicine in the group treated with reperfusion therapy.
